# Telemonitoring Tools for Glaucoma Patients: A Systematic Review of Current Trends and Applications

**DOI:** 10.3390/jcm14103317

**Published:** 2025-05-09

**Authors:** Jeniffer Jesus, Catarina Aguiar, Dália Meira, Ignacio Rodriguez-Una, João M. Beirão

**Affiliations:** 1 Unidade Local de Saúde Entre o Douro e Vouga, 4520-211 Santa Maria da Feira, Portugal; 2 Unidade Local de Saúde Gaia e Espinho, 4405-678 Vila Nova de Gaia, Portugal; 3 Instituto Oftalmológico Fernández-Vega, 33012 Oviedo, Spain; 4 Unidade Local de Saúde de Santo António, 4099-001 Porto, Portugal

**Keywords:** glaucoma, teleophthalmology, telemonitoring, digital health, telehealth

## Abstract

**Background/Objectives**: In 2010, approximately 60.5 million people were affected by glaucoma, making it the leading cause of permanent vision impairment globally. With the rise of telehealth tools and technological advancements in glaucoma care, this review aims to provide an up-to-date analysis regarding remote monitoring systems in glaucoma management. **Methods**: A systematic literature search (in compliance with PRISMA guidelines) was conducted across six databases (CINAHL, MEDLINE, PsycINFO, Web of Science, Scopus, and Cochrane Library) and one grey literature source (Google Scholar), covering the period from 2000 to 2024. Relevant studies meeting predefined inclusion criteria were identified and analyzed. **Results**: The search identified 21 eligible studies focusing on various glaucoma telemonitoring tools. Several studies demonstrated the potential for continuous remote intraocular pressure (IOP) monitoring and highlighted the effectiveness of home-based visual field-testing technologies (e.g., Melbourne Rapid Fields, Eyecatcher, and VF-Home), which showed results closely matching in-clinic tests. All 21 studies underwent risk of bias assessment with appropriate tools based on study design, and none showed a high overall risk of bias, indicating robust methodology. **Conclusions**: Glaucoma telemonitoring tools are feasible and cost-effective, helping to reduce patient travel and waiting times and improving patient satisfaction. However, periodic in-person examinations remain necessary to optimally monitor disease progression and adjust treatments. Future directions should focus on interdisciplinary collaboration and the development of advanced algorithms (including artificial intelligence) to further enhance patient outcomes in teleglaucoma care.

## 1. Introduction

Glaucoma is the second leading cause of irreversible blindness worldwide, with an estimated 76 million patients in 2020, projected to reach 111.8 million by 2040 [1]. Early detection and regular clinical follow-ups for intraocular pressure (IOP) monitoring and optic nerve imaging are crucial for slowing glaucoma progression [2]. This typically requires frequent clinical appointments for functional visual field testing and timely treatment adjustments.

In 2018, the Association of American Medical Colleges predicted that the scarcity of physicians in the United States would become even more severe, with a projected shortfall of between 33,800 and 72,700 specialists by the year 2030 [3,4,5]. Consequently, each ophthalmologist will need to manage a larger patient load, potentially leading to overbooked clinics, long patient wait times, and overcrowded waiting rooms [6,7]. As a result, patient care may suffer due to the extended intervals between appointments [7]. Recognizing this challenge, the medical community has increasingly adopted telemedicine approaches, such as the development of teleglaucoma programs and applications, into clinical practice. Therefore, this systematic review has been designed to highlight the telemonitoring tools that have been used for glaucoma over the past years, and to discuss the current and the future challenges in remote glaucoma care.

### Objectives

To provide a comprehensive review assessing the effectiveness of telemonitoring tools in glaucoma management.

To analyze the technologies used in glaucoma care.

To evaluate the impact of telemonitoring technologies on patient outcomes and disease management.

To identify future direction in glaucoma telemonitoring.

## 2. Methods

### 2.1. The Search Strategy

The search strategy was designed to identify relevant studies addressing the research question across multiple databases. The search approach followed the Preferred Reporting Items for Systematic Reviews and Meta-Analyses (PRISMA) criteria to ensure transparency and accessibility. A comprehensive search was performed in six electronic databases (CINAHL, MEDLINE, PsycINFO, Web of Science, Scopus, and the Cochrane Library) and additionally in Google Scholar (for grey literature). The search included articles published from 2000 onward. Medical Subject Headings (MeSH) and keywords used were “Home monitoring AND Glaucoma” and “Telemedicine AND Glaucoma”. Boolean operators (AND) was employed to combine search terms and refine results. Manual searches of references from included studies were also conducted to ensure comprehensive coverage of relevant literature. The review’s PICO (Population, Intervention, Comparison, and Outcome) elements are outlined in Table 1 below.

### 2.2. Inclusion Criteria

1. Studies involving patients diagnosed with glaucoma and utilizing telemonitoring tools for disease management.
2. Research evaluating quality of life or patient satisfaction using validated instruments in the context of telemonitoring.
3. Studies reporting clinical outcomes, such as disease progression, IOP fluctuations, compliance, usability, or patient-reported outcomes with telemonitoring.
4. Study designs including observational studies (cross-sectional, case–control, cohort), prospective studies, randomized controlled trials (RCTs), and feasibility studies related to telemonitoring.
5. Articles published in English with no restrictions on geographic location or healthcare settings.
6. Studies whose participants were adults (≥18 years old) at the time of the telemonitoring intervention.

### 2.3. Exclusion Criteria

1. Studies focusing on ocular conditions other than glaucoma.
2. Studies focused on in-clinic monitoring without home monitoring components.
3. Studies not directly related to glaucoma telemonitoring tools (e.g., general telemedicine not specific to glaucoma).
4. Articles with insufficient methodological quality or incomplete data.
5. Publication types that are not primary research (e.g., systematic reviews, meta-analyses, case reports, editorials, conference abstracts).

### 2.4. Data Extraction and Analysis

The screening process consisted of two stages. Initially, both the title and the abstract were reviewed under scrutiny for evaluation. This process was followed by the collecting of full-text publications of a particular selection of available research. The full-text articles were again examined at this point to code them according to the inclusion and exclusion criteria and to objectively determine their eligibility.

### 2.5. Study Selection

The search strategy for this systematic literature review was designed to capture a wide range of research on telemonitoring tools in the glaucoma population. Searches were conducted and reviewed independently by two authors (initials of authors: J.J. and J.M.B.), ensuring comprehensive coverage. In cases of disagreement, a third author (C.A./D.M./I.R.) resolved the conflicts. Initially, a broad search was conducted on several scholarly databases and registers, such as CINAHL, MEDLINE, PsycINFO, Web of Science, and Scopus, to identify all potentially relevant studies. A total of 87 articles were identified through the initial database searches. After an initial screening process, 21 articles were removed due to duplication or lack of originality, leaving 66 sources to be further reviewed by title and abstract. The titles and abstracts of the remaining 66 records were then closely examined to determine their relevance to this study’s objectives. This step led to the exclusion of 7 articles deemed irrelevant. The next stage involved retrieving the full texts of the remaining 59 records. However, 11 reports could not be retrieved, leaving 48 for further assessment. These 48 reports were then rigorously evaluated for eligibility based on predefined criteria, which included consistency in data sources, relevance to this study’s objectives, and completeness of data.

**Figure 1:**
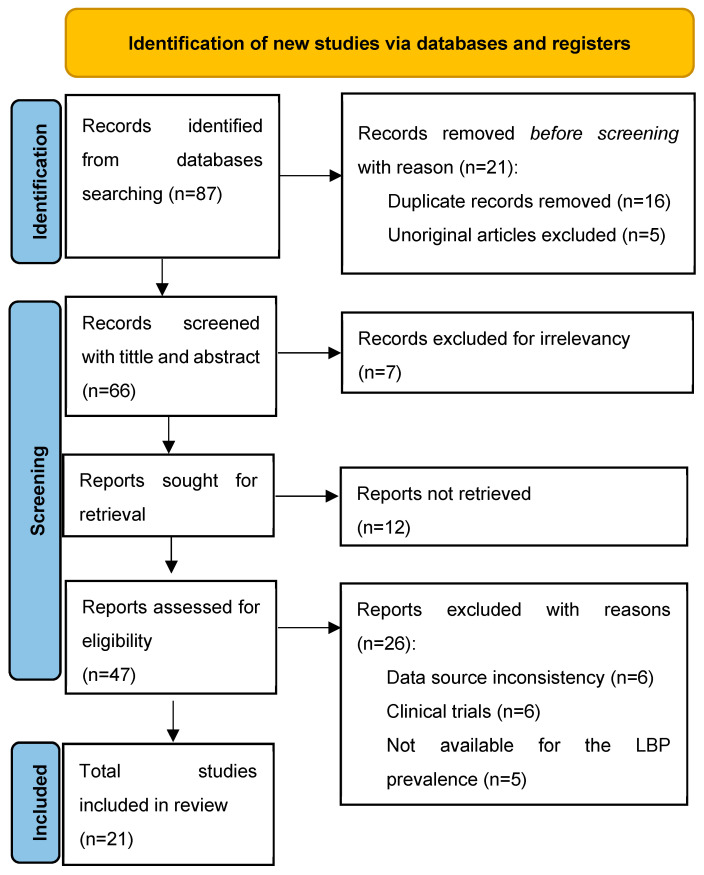
PRISMA flow diagram detailing the identification, screening, and final inclusion of studies.

Of these, 26 were excluded for reasons such as data source inconsistency, a focus on clinical trials rather than telemonitoring, irrelevance to glaucoma prevalence, lack of availability for the target objective, and data incompleteness. Ultimately, 21 studies met all eligibility criteria and were included in the review. These studies will serve as the foundation for analyzing the effectiveness of telemonitoring tools in managing glaucoma, evaluating the technologies used, and assessing the impact on patient outcomes over recent years. Figure 1 presents the PRISMA flow diagram of the study selection process, illustrating identification, screening, eligibility, and inclusion of studies. The main characteristics and findings of the included studies are summarized in Table 2.

## 3. Results

In this systematic review, 21 studies examining the use of telemonitoring tools in glaucoma patients were identified and included. These studies varied in design (prospective, observational, feasibility, cross-sectional, and retrospective) and were conducted across different geographical regions, (Europe, America, Asia, and Australia). A variety of telemonitoring tools were evaluated, such as home visual field test platforms, virtual reality-based vision tests, and home IOP monitoring devices.

Several studies such as those by Mali et al. (2023) [26] and Jones et al. (2021) [19] demonstrated how the use of home-based monitoring tools allows for earlier identification of disease progression. Home-based telemonitoring technologies were found to be feasible and effective in managing glaucoma, providing reliable data that often correlated well with in-clinic measurements.

Some studies explored the potential of continuous remote IOP monitoring. For example, studies conducted by Moraes et al. (2016) [11] and Rosenfeld et al. (2020) [17], demonstrated that continuous IOP monitoring using devices like telemetric sensors and rebound tonometers could detect IOP fluctuations and peaks more accurately than periodic in-clinic examinations. One study by Koutsonas et al. (2018) [13] showed the practicability of automated noncontact IOP monitoring by implanting a telemetric sensor at the ciliary sulcus which was well tolerated by patients and allowed 24 h recurrent measurements. Similarly, Rosenfeld et al. (2020) [17] observed that 50% of their subjects had their treatment modified due to accurate assessments provided by home monitoring with the iCare ONE rebound tonometer which also effectively detected IOP fluctuations and peaks. Moreover, studies by Levin et al. (2022) [24] and Mali et al. (2023) [26] discovered that home IOP monitoring facilitated the detection of peaks and fluctuations that were not detected during in-clinic visits, enabling timely treatment adjustments and improved patient outcomes. Additionally, Levin et al. (2022) [24] and Kadambi et al. (2023) [25] demonstrated how these technologies improve care by providing more detailed or continuous monitoring leading to better-informed clinical decisions.

The review also focused on visual field monitoring with technologies such as Melbourne Rapid Fields (MRF), Eyecatcher, and Visual Field (VF)-Home, which are known to have a high correlation with in-clinic results.

Beauregard et al. (2000) [8] showed that compression of optic nerve head images did not degrade image quality, supporting the use of compressed images in telemedicine for glaucoma diagnosis. Increasing the frequency of visual field tests through home monitoring significantly improved the early detection of rapid visual field loss among glaucoma patients (Anderson et al., 2017) [12]. For instance, Prea et al. (2020) [16] and Jones et al. (2021) [20] noted that tablet-based VF-Home and Eyecatcher visual field monitoring tools were feasible and reliable for home use with good patient adherence and minimal interference from external factors.

Despite the overall positive findings, some practical limitations were reported across the studies. The studies highlighted the challenges of self-measurement in home tonometry (Berneshawi et al., 2024) [28] and the moderate agreement between telemedicine devices and conventional tools (Kumar et al., 2007) [9]. Dave et al. (2024) [27] noted that some patients might feel concerned about interpreting their own results in the absence of a medical professional.

Nonetheless, all the reviewed studies support the notion that telemonitoring is a valuable complement to traditional face-to-face treatment methods, offering superior observational capabilities and allowing timely personalized strategies to improve outcomes for different patients.

### 3.1. Meta-Analysis Results

To further quantify outcomes, we performed meta-analyses on subsets of studies that had comparable quantitative data. Two separate meta-analyses were conducted: one evaluating mean IOP reduction in patients monitored at home versus in clinic, and another assessing visual field progression in home-monitored versus clinic-monitored patients.

### 3.2. IOP Reduction (Home vs. Clinic)

**Figure 2:**
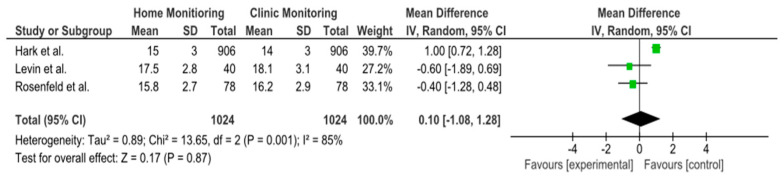
Analysis of the forest plot for mean IOP reduction. Forest plots illustrating the mean difference (MD) in IOP reduction between home- and clinic-monitored patients. The overall MD is 0.10 mmHg (95% CI: −1.08 to 1.28), indicating no significant difference between the two monitoring methods. High heterogeneity (I^2^ = 85%) was observed, leading to the use of a random-effects model. Individual study results (Hark et al., Rosenfeld et al., Levin et al.) are shown with their respective confidence intervals [14,17,24].

**Figure 3:**
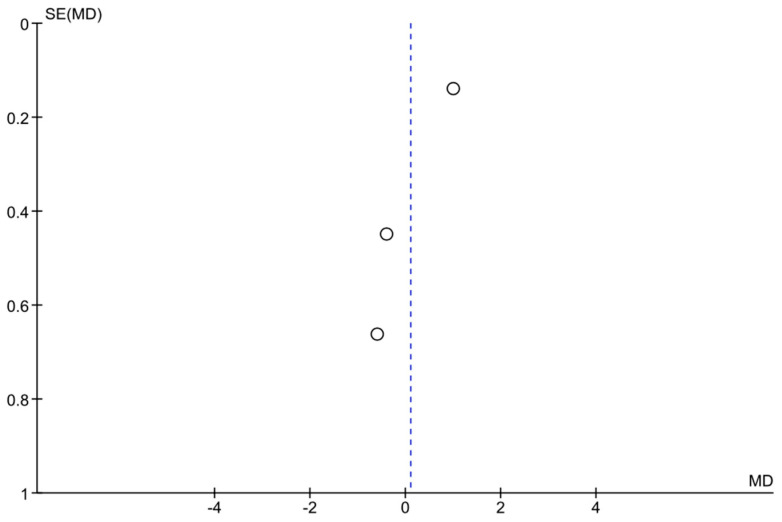
Funnel plot assessing publication bias in the meta-analysis. Each point represents a study. The *x*-axis represents the mean difference (MD), while the *y*-axis shows the standard error (SE) of MD. The symmetry of the plot suggests a low likelihood of publication bias, although interpretation should be cautious given the limited number of studies.

Three studies (Hark et al. 2018, Rosenfeld et al. 2020, and Levin et al. 2022 [14,17,24]) provided data on IOP reduction over time with home monitoring compared to clinic monitoring. The pooled analysis showed an overall mean difference (MD) of approximately +0.10 mmHg (95% confidence interval: −1.08 to +1.28 mmHg) in favor of home monitoring. This difference was not statistically significant, as the confidence interval crossed zero and the *p*-value was 0.87. In other words, there was no clear advantage of either home or clinic monitoring in terms of reducing IOP—both approaches achieved similar IOP control on average. Notably, heterogeneity among these studies was high (I^2^ = 85%), indicating substantial variability in study results. A random-effects model was used due to this heterogeneity. None of the individual studies showed a significant difference on their own: one study (Hark et al. [14]) observed a slight trend favoring home monitoring, whereas the others showed slight trends toward clinic or no difference, but all confidence intervals overlapped zero. Figure 2 illustrates the forest plot for this analysis, and Figure 3 shows the corresponding funnel plot (which did not reveal strong evidence of publication bias given the symmetry, though the number of studies was small). Overall, this meta-analysis suggests that home monitoring and clinic monitoring achieved equivalent outcomes in terms of lowering IOP. Given the high between-study variability, further research with standardized methods is needed to determine if any specific subgroups might benefit more from one approach or the other.

### 3.3. Visual Field Progression (Home vs. Clinic)

**Figure 4:**
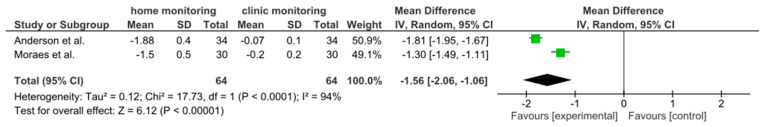
Forest plot for meta-analysis of visual field (VF) progression (mean deviation change) in home vs. clinic monitoring groups. A negative mean difference (−1.56 dB, 95% CI: −2.06 to −1.06) indicates that the home-monitored group had greater VF loss than the clinic-monitored group. The result was statistically significant (*p* < 0.00001). Studies are represented by squares proportional to their weight, and the diamond shows the overall effect. High heterogeneity (I^2^ = 94%) suggests considerable differences between studies [11,12].

**Figure 5:**
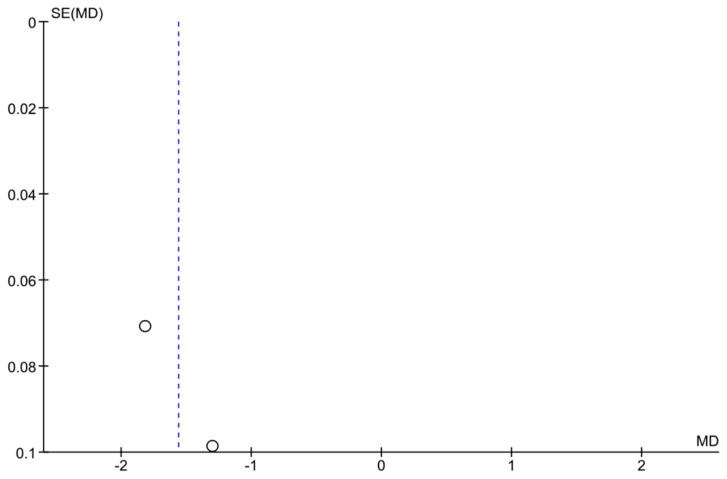
Funnel plot for the VF progression meta-analysis. The plot displays each study’s effect size (MD in VF change) versus its standard error. Some asymmetry is observed, but with only two studies, it is difficult to draw conclusions about publication bias. The limited data emphasize the need for more studies on VF outcomes in telemonitoring.

Two studies (Anderson et al. 2017 and De Moraes et al. 2016 [11,12]) provided longitudinal data on visual field loss rates in home-monitored versus clinic-monitored groups. The meta-analysis found an overall mean difference in visual field mean deviation of −1.56 dB (95% CI: −2.06 to −1.06), favoring clinic monitoring. This result was statistically significant (the confidence interval did not cross zero, *p* < 0.00001). The negative MD indicates that patients in the home monitoring groups experienced greater visual field deterioration (more negative change in dB) than those monitored with regular in-clinic exams. Figure 4 shows the forest plot for this analysis. However, heterogeneity was very high (I^2^ = 94%), reflecting differences between the two studies’ results. A random-effects model was applied for this analysis as well. Despite the small number of studies, the combined evidence suggests that, in these samples, home monitoring was less effective than frequent clinic visits in slowing visual field loss. This implies that patients monitored primarily at home had faster visual field progression, on average, than those receiving regular office-based visual field tests. It should be noted that both included studies involved relatively intensive monitoring schedules (weekly home tests in one study vs. routine care in another). Given the limited data and high variability, this finding should be interpreted with caution. Further standardized studies are required to confirm whether clinic monitoring truly confers an advantage in preserving visual fields, or if confounding factors influenced these results (Figure 4 and Figure 5).

### 3.4. Risk of Bias Assessment

All 21 studies underwent risk of bias assessment using sing appropriate tools selected by study design. Specifically, two cross-sectional studies were assessed with the AXIS tool, two case series with the JBI tool, two qualitative studies with the CASP checklist, ten diagnostic accuracy studies with QUADAS-2, and five non-randomized interventional studies with ROBINS-I. Table 3 below summarizes the bias assessment outcomes by study type.

**Figure 6:**
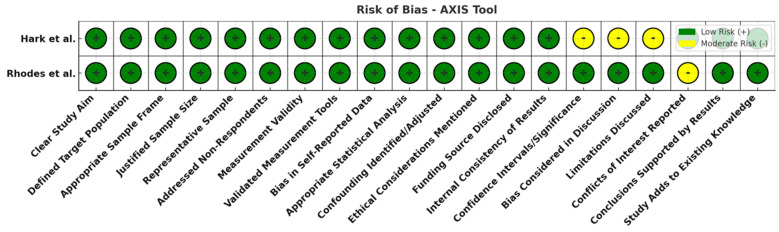
Risk of bias overview for cross-sectional studies (AXIS tool). Most domains are green (low risk), with a few yellow flags (moderate risk) for items like discussion of study limitations and statistical analyses. No high-risk (red) domains were observed, indicating reasonably sound methodology in the cross-sectional studies [14,15].

**Figure 7:**
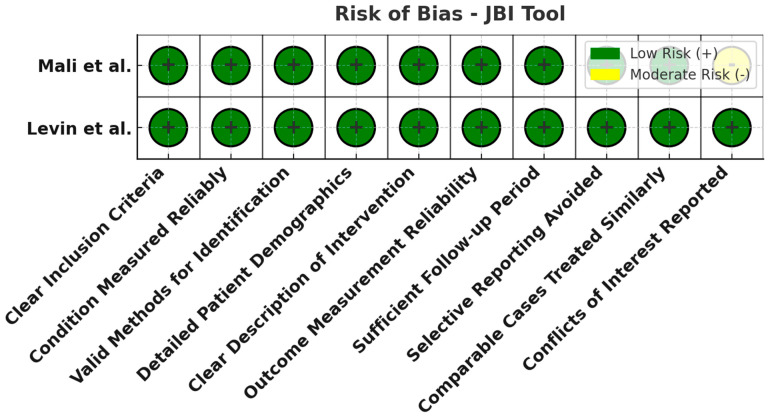
Risk of bias assessment using the JBI tool for case series. All evaluated domains are low risk (green) for both case series studies. This implies the case series had clear inclusion criteria, appropriate measurements, and complete follow-up, with no notable sources of bias [24,26].

**Figure 8:**
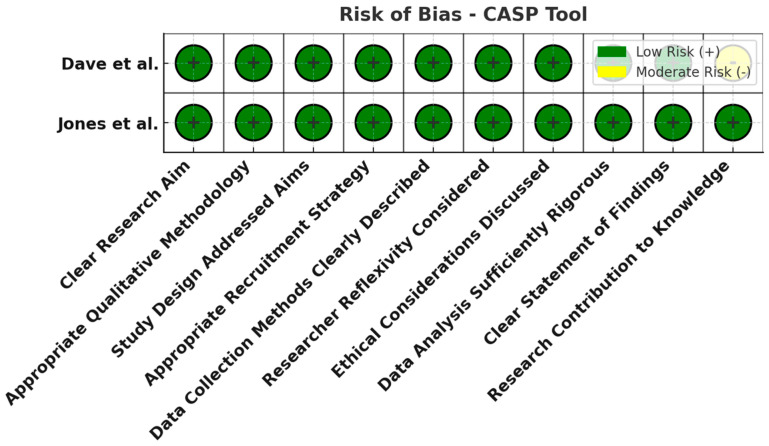
Risk of bias assessment using the CASP tool for qualitative studies. All domains (research design, recruitment strategy, data collection, reflexivity, ethical considerations, analysis, etc.) were judged low risk (green). The qualitative studies were well designed, lending credibility to their patient-reported insights [20,27].

**Figure 9:**
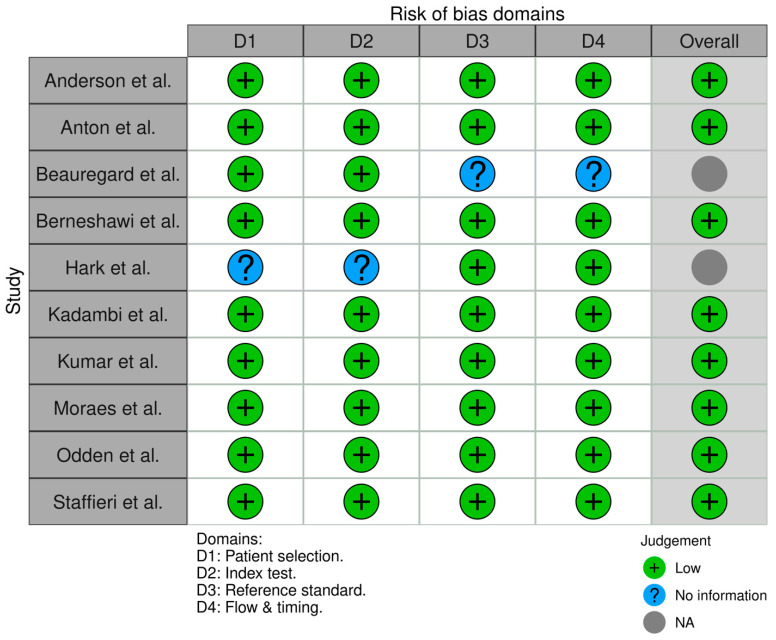
Risk of bias assessment for diagnostic accuracy studies (QUADAS-2). Most domains across the ten studies are low risk (green) in patient selection, index test conduct, and reference standard application. Some domains are marked unclear (yellow) due to insufficient information (e.g., unspecified blinding procedures). Notably, no high-risk issues (red) were identified, meaning no major biases were evident despite a few reporting ambiguities [8,9,11,12,14,21,22,23,25,28].

**Figure 10:**
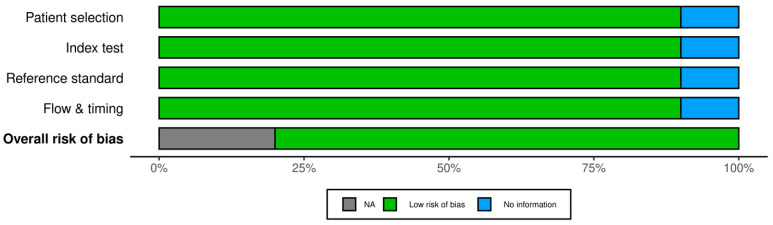
Summary of risk of bias for diagnostic accuracy studies. This figure aggregates the QUADAS-2 assessments: it shows that the majority of domains are low risk, with a portion unclear and virtually none at high risk. The unclear areas suggest where future studies should report methods more transparently (for example, details on patient flow and test interpretation).

**Figure 11:**
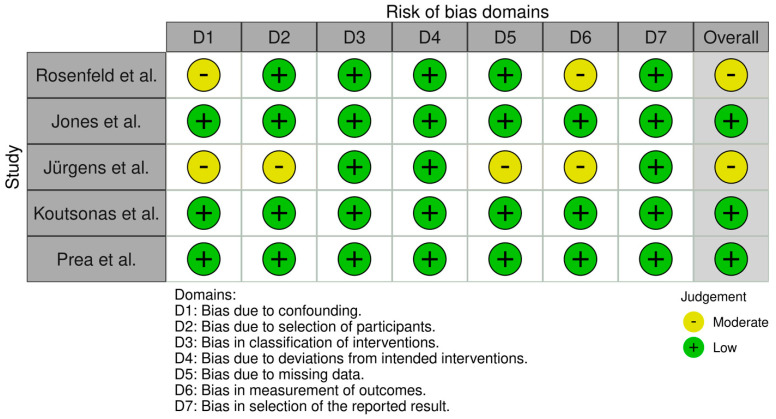
Risk of bias per domain for non-randomized interventional studies (ROBINS-I). This highlights moderate risk (yellow) in specific domains like confounding and missing data for some studies. Other domains (selection of participants, classification of interventions, outcome measurement) are largely low risk (green). There were no high-risk (red) ratings, implying that while these studies have some bias due to study design limitations, their findings are still generally credible [10,13,16,17,19].

**Figure 12:**
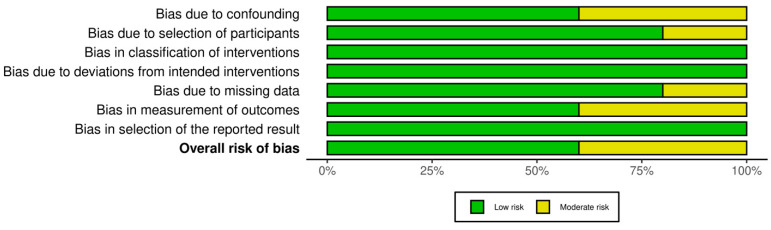
Summary of overall risk of bias for non-randomized interventional studies (ROBINS-I). This summary underscores that none of the five studies had a critical or high overall risk; most domains are low risk with a few moderate. The results support the validity of these studies’ conclusions while acknowledging the need to interpret certain outcomes (e.g., those susceptible to confounding) with caution.

In summary, the risk of bias assessment indicated that the majority of included studies were methodologically robust, with no study having a high overall risk of bias. A few domains in certain studies showed moderate or unclear risks, but these were isolated concerns. The comprehensive application of multiple bias assessment tools (Figure 6, Figure 7, Figure 8, Figure 9, Figure 10, Figure 11 and Figure 12) provides a transparent overview of study quality, supporting confidence in the reliability of the review’s findings.

## 4. Discussion

Telemonitoring tools are considered some of the most important developments in managing chronic conditions, such as glaucoma, where continuous monitoring and real-time data are essential. These technologies enable more frequent assessment of glaucoma status outside of the clinic, which can potentially lead to earlier interventions and more personalized care. However, the adoption of telemonitoring must consider certain limitations identified in the studies, such as technical challenges and patient comfort with technology. One clear benefit of glaucoma telemonitoring is its ability to augment traditional care. All reviewed studies concur that telemonitoring should be used as a supplement to, not a replacement for, in-person care. Telemonitoring provides additional data between clinic visits, offering a more comprehensive view of a patient’s condition. The evidence from this review shows that home-obtained data (IOP measurements, VF tests, etc.) can closely mirror clinical data in reliability, thereby helping clinicians detect meaningful changes in disease status sooner. For example, home tonometry devices captured IOP spikes that were missed during daytime office hours, and frequent home VF tests caught rapid progression that might have gone undetected with infrequent clinic testing. Such continuous monitoring empowers ophthalmologists to make timelier and better-informed treatment decisions.

At the same time, the limitations and patient concerns observed must be addressed for telemonitoring to be effectively integrated. Some patients experienced technological issues (device malfunctions, internet connectivity problems, difficulty using the devices) and reported anxiety when interpreting their own results without immediate professional feedback. This suggests a need for robust technical support and patient education as part of telemonitoring programs. Additionally, the moderate discrepancies noted between certain telemetric measurements and gold-standard clinical measurements (e.g., home iCare tonometry vs. GAT) indicate that current telemonitoring tools, while useful, may not yet perfectly replicate clinic assessments in all cases. Patients also worry that telemedicine might reduce face-to-face contact with their doctor, potentially affecting the quality of care. Therefore, clinicians should reassure patients that telemonitoring is intended to complement regular visits—enhancing monitoring frequency and convenience, but not eliminating in-person evaluation when needed.

The following discussion section provides an overall summary and evaluation of these tools, including a description of the technologies used, an assessment of their impact on patient outcomes, and an exploration of future trends in glaucoma telemonitoring.

### 4.1. Efficiency of Telemonitoring Tools in Glaucoma Management

The findings of this review highlight that telemonitoring tools can enhance glaucoma management efficiency by providing continuous, real-time data on IOP and visual function. In multiple studies, home-based measurements closely aligned with in-clinic measurements of IOP and visual fields, demonstrating that these tools can reliably extend monitoring beyond the clinic. The ability to track IOP fluctuations in real-time is particularly valuable: telemetric IOP sensors and home tonometers captured significant diurnal IOP variations and peak pressures that are often missed during routine daytime visits. Early recognition of these IOP changes at home provides insights that allow for timely interventions, such as medication adjustments or scheduling earlier appointments, which may help halt or slow disease progression. Home visual field monitoring devices similarly improved the efficiency of care by enabling more frequent functional testing. As shown in Anderson et al. (2017) [12], weekly home VF tests led to earlier detection of rapid VF loss compared to infrequent clinic tests. By increasing test frequency in a convenient home setting, telemonitoring can uncover deterioration sooner, prompting quicker therapeutic responses. This is especially important in glaucoma, where early detection of progression can significantly influence the course of treatment. Overall, the studies indicate that integrating these home-based tools with standard care provides a more continuous and detailed picture of a patient’s condition, thereby improving the timeliness and potentially the quality of interventions. Another aspect of efficiency is the reduction in unnecessary clinic visits. Telemonitoring, by providing data remotely, can help identify which patients are stable (and can avoid an immediate clinic visit) and which patients show signs of instability (and need prompt evaluation). Several included studies suggested that with reliable telemetric data, clinicians could stratify follow-up intensity based on actual need rather than fixed schedules. In practice, this means healthcare resources can be allocated more efficiently, focusing in-person attention on patients who require intervention while allowing stable patients to safely continue monitoring at home.

### 4.2. Analysis of Technologies Used in Glaucoma Care

This review examined a wide range of technologies employed for remote glaucoma management, each tailored to specific aspects of the disease (IOP vs. visual function). For IOP monitoring, common technologies included portable rebound tonometers (like iCare HOME) and implantable or contact lens sensors that measure IOP continuously. Devices such as the iCare HOME tonometer were frequently used for patients to self-measure IOP at home. These tools proved effective in capturing IOP patterns—importantly, they often detected IOP peaks or significant fluctuations outside of office hours. For instance, several patients in the reviewed studies experienced their highest IOP in the early morning hours, which was only discovered through home measurements. Recognizing these off-hour IOP spikes can be critical; it enabled clinicians to intensify treatment or perform interventions (such as laser or surgery) at the right time, potentially preventing optic nerve damage that might have occurred if those peaks went unnoticed. The review also covered various visual field (VF) telemonitoring tools. Examples include tablet-based perimetry apps (VF-Home), laptop-based tests (MRF), and even virtual reality platforms (Olleyes VisuALL). These tools have demonstrated encouraging reliability, with strong correlation to standard Humphrey perimetry results in clinic. Key factors for success in these VF tools were good patient compliance and user-friendly design. Indeed, ease of use is crucial: patients need to feel comfortable performing the test at home for results to be reliable. The studies noted that patient training and clear instructions contributed to high adherence rates for home VF testing (as high as 88–100% completion of scheduled tests in some trials). Overall, the technologies highlighted in this review demonstrate the diversity and maturity of available telemonitoring options. From a simple smartphone-based vision test to sophisticated implantable sensors, there is a spectrum of tools catering to different needs and contexts. This flexibility means clinicians can tailor telemonitoring strategies to individual patients—for example, using home tonometry for a patient with IOP variability, or home VF testing for a patient with progressing field loss. It is important to note, however, that while these technologies are effective, challenges remain in their implementation. Technical issues (device calibration, data transmission) and ensuring patient proficiency with devices are areas for improvement. Continued evolution of telemonitoring technology, with a focus on user-centric design and reliability, is expected to further integrate these tools into routine glaucoma care.

### 4.3. Impact of Telemonitoring Technologies on Patient Outcomes and Disease Management

Telemonitoring technologies have shown a meaningful impact on patient outcomes by advancing the early detection and proactive management of glaucoma. Early detection is critical in glaucoma to prevent irreversible vision loss, and telemonitoring directly supports this by increasing the frequency and scope of monitoring. By enabling measurements to occur in a patient’s daily environment, telemonitoring ensures that changes in a patient’s condition (such as IOP spikes or rapid VF deterioration) are detected promptly rather than at a later scheduled visit. This more dynamic tracking of the disease allows clinicians to adjust treatment plans sooner—for example, adding or intensifying medications or recommending surgical intervention before significant progression occurs.

The continuous stream of data from telemonitoring also enhances the precision of glaucoma management. Real-time IOP and VF information provide a deeper understanding of each patient’s disease behavior. For instance, trends gleaned from home IOP readings can reveal if a patient is experiencing greater pressure variability, which is a risk factor for progression. Clinicians can then personalize the therapy (perhaps by switching to a medication that provides round-the-clock IOP control or scheduling a pressure-lowering procedure) to address those specific patterns. Similarly, consistent home VF testing can help differentiate true progression from test variability by analyzing multiple data points in a shorter period. The studies reviewed showed that this personalized, data-driven approach can improve long-term outcomes by aligning treatment decisions more closely with the patient’s current status, rather than relying on infrequent snapshot assessments at clinic visits.

Importantly, telemonitoring can also positively affect patient behavior and empowerment. Several qualitative findings indicated that patients felt more engaged in their own care when using telemonitoring tools. Having the ability to measure their own IOP or perform their own VF test gave patients a sense of control and responsibility in managing their glaucoma. This increased engagement can translate to better adherence to treatment (patients who regularly check their vision or pressure might be more vigilant in using their eye drops or following medical advice). Furthermore, seeing real-time feedback about their condition can motivate patients to maintain healthy behaviors and follow-up as needed. For example, if a patient notices their home readings trending worse, they may be more inclined to take medications regularly or report issues promptly.

On the other hand, the impact of telemonitoring is not universally positive without addressing some concerns. A few studies highlighted that some patients might feel overwhelmed by continuous surveillance or the responsibility of self-testing. The psychological impact of constant monitoring (sometimes termed the “worried well” phenomenon) means that while many patients feel empowered, a subset may experience anxiety. This underscores that telemonitoring programs should include adequate counseling and easy access to professional guidance when patients have questions or alarming readings, to mitigate anxiety.

In summary, telemonitoring has the potential to transform glaucoma management from a model of periodic interventions to one of continuous, individualized care. By improving the timeliness of interventions and involving patients more directly in monitoring, these technologies can help preserve vision and quality of life. The overall patient outcomes can improve not just in terms of clinical measures (like slower progression rates) but also in patient satisfaction and confidence in managing their disease.

### 4.4. Future Directions in Glaucoma Telemonitoring

The future of glaucoma telemonitoring is at a turning point as many areas can be developed, which will result in significant improvements. One key direction is the adaptation of existing technology to overcome challenges such as technical limitations and patient concerns over tool usability. Consequently, integrating machine learning and artificial intelligence methods into telemonitoring has the potential to improve sensitivity and reliability considerably. For instance, Jones et al.’s (2021) [19] study demonstrated how AI could be used to enhance the interpretation of home monitoring data, reducing errors while maintaining user-friendliness. Because of this, AI algorithms could be used to analyze the collected data by these tools, identify patterns, and provide predictive insights that can guide treatment decisions. Also in the technological field, it is crucial to develop robust infrastructure to improve and maintain data security and patient privacy.

Additionally, in this study, most of the reviewed articles focused on IOP and VF monitoring, creating a need to explore other aspects of glaucoma management, such as the monitoring of optic nerve health, visual acuity, patient-reported symptoms, quality of life and outcomes. This would provide a more holistic approach in future to telemonitoring and ensure that all aspects of the disease are adequately addressed.

Another critical path forward involves improving the accessibility and affordability of glaucoma telemonitoring. As these instruments become more sophisticated, it is important to ensure that they remain accessible, particularly for patients in underserved communities or with limited resources. This may require the development of more affordable products and hybrid models that integrate telemonitoring with traditional in-clinic visits. This will be crucial because expanding the reach of telemonitoring technologies will help bridge gaps in healthcare access, allowing more patients to benefit from continuous, personalized monitoring. Furthermore, patient education and support systems should be strengthened to alleviate concerns and enhance the adoption of these tools, ensuring that patients feel confident and comfortable using them as part of their daily routine.

While the previously published studies highlighted the potential benefits of telemonitoring especially in improving early detection and disease control, there is still a need for the investigation of long-term impacts on healthcare costs, disease progression and overall health outcomes.

Comprehensive studies could help researchers and physicians to better understand how telemonitoring affects patient well-being over time and the underlying economic implications of its large-scale adoption. This understanding will be crucial when considering the incorporation of telemonitoring into standard care practices.

## 5. Conclusions

The utilization of telehealth in ophthalmology has increased dramatically in the past decade, accelerated in part by the COVID-19 pandemic. The introduction of telemonitoring tools in glaucoma management represents a significant advancement, enabling more continuous and personalized care for glaucoma patients. This systematic review evaluated the efficiency of telemonitoring tools in glaucoma management, analyzed the technologies employed, assessed their impact on patient outcomes, and identified future directions for telemonitoring in glaucoma. By synthesizing evidence from 21 studies across various countries and settings, the review highlights the substantial potential of telemonitoring tools to improve and possibly revolutionize glaucoma care.

The primary objectives of this review were to comprehensively assess current telemonitoring tools for glaucoma, explore the technologies used, and evaluate their impact on patient outcomes. The findings demonstrate that telemonitoring tools are both feasible and effective in providing continuous, reliable data that correlate well with in-clinic measurements. In line with our objectives, one key finding is the valuable role of continuous IOP monitoring: home tonometry devices can detect IOP fluctuations and peaks that often go unnoticed during routine clinic visits. This supports the notion that telemonitored IOP data can enhance clinical decision making by revealing occult risk factors for progression. Another major finding is the promise shown by home visual field monitoring. Tools such as MRF, Eyecatcher, and VF-Home exhibited high correlation with standard in-office perimetry, proving to be reliable and effective for home use. Notably, increasing the frequency of VF testing through home monitoring significantly improved the early detection of rapid VF loss in glaucoma patients. High patient adherence and minimal interference from external factors were reported for these home VF platforms, strengthening the case for their use in regular monitoring.

The implications of these findings for glaucoma management are considerable. The ability to continuously monitor IOP and visual fields at home offers the potential for more frequent assessments and timely interventions, leading to more personalized treatment plans. Telemonitoring provides clinicians with a more comprehensive understanding of a patient’s condition between visits, resulting in better-informed decision making and potentially improved long-term outcomes. One notable implication is the potential of telemonitoring to reduce the burden on healthcare facilities: if patients can reliably monitor their condition at home, the frequency of routine clinic visits could be reduced for stable patients, freeing up clinic time and resources for more urgent or complex cases.

This is particularly relevant given the rising prevalence of glaucoma worldwide and the need for scalable management strategies. Telemonitoring can also improve patient engagement and empowerment in managing glaucoma. Qualitative evidence indicated that patients appreciate the convenience and sense of control these tools offer. By involving patients more directly in monitoring their condition, telemonitoring may enhance adherence to treatment regimens and encourage a proactive approach to disease management.

In essence, patients become active partners in care, which can positively influence outcomes.

Despite these benefits, the review also highlighted challenges associated with telemonitoring. Technological issues (such as device reliability and data management logistics) and the potential for increased patient anxiety due to continuous self-surveillance were noted as concerns. Addressing these challenges is crucial for successful integration of telemonitoring into routine practice. This includes ensuring that devices are user-friendly and dependable, providing patients with support and clear guidelines on interpreting their results, and reassuring patients that telemonitoring is meant to augment rather than replace in-person care. Further research is needed to fill the gaps identified in this review. Future studies should aim to expand the scope of telemonitoring (e.g., incorporating optic nerve imaging at home), improve technology accessibility, evaluate long-term outcomes of telemonitored patients, address patient concerns more thoroughly, and explore the incorporation of artificial intelligence for data analysis. One limitation of our review is the heterogeneity of interventions and outcomes among included studies, which made direct comparisons challenging. However, since our analysis was largely narrative, this heterogeneity did not hinder us from drawing overall conclusions. It remains advisable that patients with severe or uncontrolled glaucoma, or those with new alarming symptoms, continue to have prompt face-to-face evaluations to avoid missing critical issues that telemonitoring might not capture.

In conclusion, as technology continues to evolve, telemonitoring is expected to play an increasingly central role in glaucoma management. Telemonitoring has the potential to revolutionize the approach to this chronic condition by shifting care from episodic clinic visits to a model of continuous, personalized monitoring. A telehealth-enhanced strategy can reduce the overall burden on healthcare systems and improve patient outcomes and satisfaction. Telemonitoring should be viewed as a powerful complement to traditional clinical practice—one that, when implemented thoughtfully, can significantly enhance the quality and efficiency of glaucoma care.

## Figures and Tables

**Table 1:** The PICO Framework.

Element	Keywords
Population	Glaucoma patients (adult population), requiring ongoing monitoring of a chronic condition.
Intervention	Telemonitoring tools (e.g., contact lens IOP sensors, home-based visual field tests, portable IOP monitors).
Comparison	Traditional monitoring and standard in-person follow-up care.
Outcome	Continuous monitoring, early detection of changes, treatment adherence, clinical outcomes, accessibility, convenience, patient and provider satisfaction, cost-effectiveness, and EHR integration.

**Table 2:** Data extraction table.

SR #	Author	Year	Country	Study Design	Sample Size	Population Characteristics	Measurement Tools	Findings
1	Beauregard et al. [8]	2000	USA	Experimental study	302 slides of 155 patients	20 normal patients, 55 glaucoma suspects, 80 glaucoma patients.	Slide scanner (PhotoSmart, Hewlett Packard), TIFF and JPEG formats, Thumbsplus 3.0 software, standardized reticule for planimetry, millimeter ruler, t-tests, regression analysis	Compression did not degrade the quality of optic nerve head images. Both TIFF and JPEG formats produced similar results. Repeatability was better for digital images.
2	Kumar et al. [9]	2007	Australia	Comparative analysis	399 eyes	General population with glaucoma risk.	Telemedicine-friendly devices and conventional screening tools	Telemedicine devices showed good agreement with conventional tools, with 91.1% sensitivity for glaucoma detection when combined with conventional tests.
3	Jürgens et al. [10]	2012	Germany	Telemedical home monitoring study	153 patients 70 patients complete the study	The average age was 60.3 years (±9.6 years). 33 patients were female, and 37 were male.	Home monitoring system for IOP and BP	The study revealed significant circadian fluctuations in ocular perfusion pressure (OPP) in patients with primary open-angle glaucoma. OPP and blood pressure were lowest in the morning, while intraocular pressure was significantly lower in the evening.
4	Moraes et al. [11]	2016	Sweden	Prospective, cross-sectional study	40 treated glaucomatous patients	Patients had 8 or more 24-2 visual field tests.	Visual field MD	The rate of visual field mean deviation (MD) change was slower at the time of contact lens sensor (CLS) recording compared to before, indicating a deceleration in disease progression. Key predictors of faster progression included the number of long peaks and the mean peak ratio during wakefulness. The CLS parameters provided more accurate predictions of glaucoma progression than traditional Goldmann IOP measures. Thus, 24 h IOP-related parameters obtained through CLS are effective in identifying glaucomatous eyes at higher risk of progression, enhancing treatment monitoring and patient outcomes.
5	Anderson et al. [12]	2017	Australia, UK	Computer simulation, cohort study	43 patients	Mean age of participants was 71 years, with an age range of 37 to 89 years. Participants included: Patients with treated glaucoma (both open-angle and closed-angle). Individuals with ocular hypertension. Glaucoma suspects.	Melbourne Rapid Fields (MRF)	Increasing the frequency of visual field tests through home monitoring significantly improved the early detection of rapid visual field loss in glaucoma. Weekly home monitoring detected progression more quickly than biannual clinic tests, even with imperfect compliance and increased test variability. This suggests home monitoring could be a viable alternative to frequent in-clinic testing, potentially reducing clinical workload, though cost–benefit analysis is required.
6	Koutsonas et al. [13]	2018	Germany	Feasibility study	30 patients	The average age of the patients was 68.3 years. The majority of the patients were male (73%).	Telemetric intraocular pressure sensor	The study demonstrated the feasibility of automated, noncontact IOP monitoring using a novel telemetric sensor implanted in the ciliary sulcus. The system allowed for repeated 24 h measurements, with the device well tolerated by patients. The findings indicated that reliable, continuous home monitoring of IOP is possible, which could enhance glaucoma management through more consistent data on IOP fluctuations.
7	Hark et al. [14]	2019	USA	Cross-sectional study	906 participants	High-risk ethnic groups, aged >40.	Fundus photography, intraocular pressure measurements	High diagnostic confirmation (86%) between telemedicine screening and comprehensive eye exams. Telemedicine screening is adaptable for wider implementation.
8	Rhodes et al. [15]	2019	USA	Observational study	110 patients	Glaucoma patients aged 60 and above.	Life Space Questionnaire, Preferences for Telemedicine Questionnaire	71% were open to telediagnosis, and 74% to teleintervention; patient openness to telemedicine correlated with demographics, health status, and proximity to the clinic.
9	Prea et al. [16]	2020	Australia	Single-center, observational, longitudinal, compliance study	101 participants 186 Eyes Studied	88% of participants successfully completed at least 1 home examination. 69% of participants completed all 6 home examinations. Median duration between tests: 7.0 days [with an interquartile range of 7.0–8.0 days].	Tablet-based visual field monitoring (VF-Home)	VF-Home demonstrated high short-term compliance, with 88% of participants completing at least one test and 69% completing all six. Home tests showed a high correlation (R = 0.85) with in-clinic outcomes. Barriers included IT issues and lack of motivation. Overall, VF-Home provided reliable data comparable to in-clinic testing, with slightly higher fixation loss but similar false positives.
10	Rosenfeld et al. [17]	2020	Israel	Retrospective case series	40 patients 80 eyes	Mean age of 59.1 ± 14.6 years (range: 24–78 years). The study involved patients with known glaucoma or glaucoma suspects.	iCare ONE rebound tonometer	Home monitoring with RT-ONE demonstrated that mean IOP at home was significantly lower compared to clinic measurements. Peaks were observed more frequently in the morning. Home monitoring led to treatment modifications in 55% of cases, indicating it provides accurate assessment of IOP fluctuations and peaks, facilitating better treatment for glaucoma patients.
11	Hark et al. [18]	2020	USA	Cross-sectional study	906 participants	High-risk individuals aged >40.	Fundus photography, intraocular pressure measurements	17.1% of participants had unreadable images; high proportion (65.2%) diagnosed with ocular pathology in follow-up, indicating necessity for comprehensive exams.
12	Jones et al. [19]	2021	UK	Qualitative study using semi-structured interviews	20 adults	10 women and 10 men (assuming the remaining participants were male). Median age of participants was 71 years.	Eyecatcher (home-based visual field test)	Participants found VF home monitoring with Eyecatcher acceptable and identified benefits compared to the Humphrey Field Analyser (HFA). Key themes included positive comparisons with HFA, ease of use, and practicalities for wider implementation.
13	Jones et al. [20]	2021	UK	Prospective longitudinal feasibility study	20 adults	The median age of participants was 71 years. All participants had an established diagnosis of glaucoma.	Tablet-based visual field test (Eyecatcher)	The study found that monthly home monitoring of visual fields using Eyecatcher was feasible and well adhered to by patients with glaucoma. There was good concordance between home and clinic measurements. Anomalous tests could be identified using machine learning techniques. Adding home monitoring data to standard clinical tests reduced measurement error significantly. Home testing was unaffected by ambient illumination and had a median test duration of 4.5 min.
14	Staffieri et al. [21]	2021	Australia	Prospective study	211 people	First-degree relatives of glaucoma patients.	Clinical assessments by a trained nurse, graded by an ophthalmologist	5% of participants had undiagnosed glaucoma; telemedicine is effective for screening high-risk individuals, and nurses can be trained for initial examinations.
15	Odden et al. [22]	2021	USA	Real-life trial	200 patients	Adult glaucoma patients.	In-person vs. remote assessments	Agreement between in-person and remote assessments was moderate (kappa values ranged from 0.19 to 0.35), suggesting that telemedicine may be effective in monitoring progression.
16	Anton et al. [23]	2021	Spain	Stratified sample study	1006 eyes	General population.	Optical coherence tomography (OCT), retinography	Moderate to good interobserver agreement, improved with evaluator experience. OCT and retinography both effective for telemedicine glaucoma screening.
17	Levin et al. [24]	2022	USA	Retrospective case series	12 patients 18 eyes	7 POAG 1 pigmentary glaucoma 1 juvenile OAG.	iCare HOME	IOP measurements differ from in-clinic IOP measurements. Home tonometry can be used to effectively monitor *peri*-interventional patterns in IOP variability and peaks that could be affecting glaucomatous progression.
18	Kadambi et al. [25]	2023	India	Prospective observational study	70 participants were trained for the study. 51 participants (72.9%) were able to take reliable readings. 102 eyes from 51 patients were analyzed.	Age range: 18 to 80 years. Mean age: 53 ± 16 years. Glaucoma status: Patients with glaucoma and glaucoma suspects.	iCare HOME, Goldmann applanation tonometer (GAT)	Home tonometry using iCare HOME demonstrated strong correlation between optometrist- and participant-taken IOP measurements (r = 0.90, *p* < 0.0001). However, the agreement between iCare HOME and GAT was limited, with 37% of eyes showing synchronous peaks during diurnal variation testing. The study concluded that home tonometry is easy and feasible, but due to limited agreement, it cannot substitute GAT for diurnal variation testing in clinical practice.
19	Mali et al. [26]	2023	USA	Prospective observational study	29 patients were enrolled in the ILLP participation portion of the study. 28 of 29 cases successfully performed home tonometry. 22 participating families submitted data formally. 150 potential participants were surveyed, with 83 (55%) responding.	Age range of enrolled patients: 0.25 to 41 years. Mean age: 12.9 years.	iCare rebound tonometer	Home rebound tonometry was used for twice daily IOP monitoring in 29 childhood glaucoma patients. This tool prompted and/or validated glaucoma-related surgery in 55% of patients and led to medication changes in 76% of patients. Survey responses indicated that 84% of parents or patients would be interested in home tonometry, and 80% of physicians believed it would improve patient management. However, only 14% of physicians currently lend tonometers, primarily due to financial concerns.
20	Dave et al. [27]	2024	UK	Qualitative study using focus groups and questionnaires	15 people	8 participants had glaucoma (5 women, median age 74). 7 participants had age-related macular degeneration (AMD) (4 women, median age 77).	Melbourne Rapid Fields, Eyecatcher, Visual Fields Fast, Alleye, PopCSF, SpotChecks	Participants believed home monitoring could provide greater control but had concerns about it replacing face-to-face appointments, clinician burden, technological challenges, and anxiety from results. Most devices were rated highly for usability.
21	Berneshawi et al. [28]	2024	USA	Pilot study	15 participants 9 participants (60%) completed the study.	60% of enrolled participants completed the study, meaning 6 participants were excluded due to inability to self-measure using the iCare HOME device.	Olleyes VisuALL Virtual Reality Platform (VRP), iCare HOME handheld self-tonometer	Unsupervised at-home multi-day glaucoma testing showed a significant correlation between VRP and in-clinic HFA tests (r² = 0.8793, *p* < 0.001). At-home tonometry provided similar IOP values to trainer-obtained iCare HOME and in-clinic GAT measurements, but higher maximum IOP values were captured at home. A total of 60% of participants completed the study; the remaining 40% struggled with self-measuring IOP using iCare HOME. The findings suggest that at-home remote glaucoma monitoring is feasible and correlates with in-office testing.

**Table 3:** Study Bias Summary.

Study Type	Risk of Bias	Key Concerns	Overall Reliability
Cross-Sectional Studies (AXIS)	Predominantly low, with moderate concerns in a few areas	Clarity of limitations, some statistical considerations	No high-risk issues detected
Case Series (JBI)	Low risk in all evaluated domains	None	No significant concerns
Qualitative Studies (CASP)	Low risk in all domains	None	Methodologically rigorous
Diagnostic Accuracy Studies (QUADAS-2)	Low risk in key domains, some unclear risk in certain areas	Lack of sufficient detail in some domains (e.g., reference standard blinding)	Methodologically reliable despite reporting gaps
